# Serum branched chain amino acids: an effective indicator of diabetic kidney disease

**DOI:** 10.3389/fendo.2023.1269633

**Published:** 2023-11-27

**Authors:** Min Liu, Yanhui Yang, Yajin Liu, Xiaoyue Peng, Yi Hou, Xuejiao Zhang, Haipeng Sun, Chunyan Shan

**Affiliations:** ^1^ NHC Key Laboratory of Hormones and Development, Chu Hsien-I Memorial Hospital and Tianjin Institute of Endocrinology, Tianjin Medical University, Tianjin, China; ^2^ Tianjin Key Laboratory of Metabolic Diseases, Tianjin Medical University, Tianjin, China

**Keywords:** type 2 diabetes mellitus, branched -chain amino acids, keto acids, diabetic kidney disease, proteinuria

## Abstract

**Introduction:**

In recent years, there has been a growing association between elevated circulating levels of branched-chain amino acids (BCAA) and diabetes mellitus. However, the relationship between serum BCAA levels and diabetic kidney disease (DKD) remains ambiguous. This study aims to investigate serum BCAA levels in DKD patients at various stages and assess the correlation between BCAA and clinical characteristics.

**Materials and methods:**

We enrolled patients with type 2 diabetes mellitus (T2DM) who were admitted to our hospital and categorized them into three groups based on different DKD stages: normal proteinuria, microproteinuria, and macroalbuminuria groups. Forty healthy volunteers were included as the control group, and we measured serum BCAA concentrations using liquid chromatography-mass spectrometry (LC-MS). Subsequently, we conducted correlation and regression analyses to assess the associations between BCAA and clinical indicators.

**Results:**

Serum BCAA levels were significantly elevated in T2DM patients compared to healthy controls. However, these levels exhibited a gradual decline with the progression of DKD. Furthermore, after adjusting for age, gender, and disease duration, we observed an independent association between serum albumin, urinary transferrin, and urinary microalbumin with BCAA.

**Discussion:**

Our findings suggest a noteworthy decline in serum BCAA levels alongside the advancement of DKD. Additionally, serum BCAA exhibits an independent correlation with renal function indicators. These observations point to the possibility that serum BCAA concentrations in individuals with T2DM hold promise as a crucial predictor for both the initiation and progression of DKD.

## 1 Introduction

Diabetic kidney disease (DKD) is the most common microvascular complication of diabetes (1, 2); approximately 20-40% of patients with diabetes reportedly have DKD (3). As the prevalence of diabetes continues to rise globally, DKD is becoming a major public health problem and one of the leading causes of end-stage renal disease (ESRD) (3). DKD is a progressive disease characterized primarily by impaired glomerular filtration barrier function, predominant microalbuminuria, usually with few early symptoms. The urine protein-to-creatinine ratio (UACR) is commonly used clinically to assess early DKD (4, 5); as the disease progresses, patients gradually develop glomerulosclerosis and interstitial fibrosis of the renal tubules, leading to massive proteinuria and ESRD. The pathogenesis of DKD is complex, and current research indicates that insulin resistance, glucolipid metabolism disorders, long-term chronic inflammation, oxidative stress, and endothelial dysfunction are all involved in the development of DKD (6–8). The management of DKD primarily revolves around the maintenance of optimal blood glucose control. Nevertheless, it is crucial to emphasize that early diagnosis and intervention hold the potential to mitigate and postpone the onset of ESRD. Hence, the timely identification of DKD assumes paramount significance.

Branched-chain amino acids (BCAA) are amino acids characterized by their branched side chain structures, which include leucine, isoleucine, and valine. These essential amino acids cannot be synthesized within the human body and must be obtained through dietary sources. Upon ingestion, BCAAs undergo transamination facilitated by branched-chain amino acid aminotransferase (BCAT) to form corresponding branched-chain keto acids (BCKA). These BCKAs encompass α-ketoisovaleric acid (KIV), α-ketoisocaproic acid (KIC), and α-keto-β-methylvaleric acid (KMV). Subsequently, these BCKAs undergo further oxidative degradation mediated by the branched-chain keto acid dehydrogenase complex (BCKD) (9). In addition to serving as a crucial energy source for the body, BCAAs function as signaling molecules with diverse and vital biological activities, participating in a wide array of metabolic pathways. These activities are intricately regulated in both healthy and diseased states (9, 10). Research has demonstrated that BCAA levels become elevated in the circulation of obese individuals, and this elevation is strongly associated with the development of insulin resistance (10, 11). Furthermore, recent investigations have identified elevated plasma levels of 3-Hydroxyisobutyrate (3-HIB), a BCAA metabolite, as a potential predictive marker for the future risk of type 2 diabetes mellitus (T2DM) (12). Remarkably, alterations in plasma-free amino acid profiles, particularly in BCAA levels, precede the onset of T2DM and significantly correlate with future diabetes diagnosis (13, 14). Elevated concentrations of BCAAs, both in plasma and tissues, have also been observed in the context of breast cancer, concomitant with increased expression of catabolic enzymes, including branched-chain amino acid transaminase 1 (BCAT1) (15). Moreover, our research group has uncovered that defective BCAA catabolism contributes to heart failure, a condition marked by oxidative stress and metabolic disturbances induced by mechanical overload (16). Consequently, BCAAs and their derivatives hold promise as potential biomarkers for various diseases. However, despite this extensive research, there remains a noticeable gap in our understanding regarding the relationship between BCAAs and DKD. To what extent is the catabolism of BCAAs impaired in DKD? This question remains an area of limited investigation.

The aim of this study was to assess the serum levels of BCAA and BCKA in individuals diagnosed with T2DM and examine their association with the progression of DKD.

## 2 Materials and methods

### 2.1 Participants

One hundred and twenty patients with T2DM who were admitted to the Chu Hsien-I Memorial Hospital Department of Tianjin Medical University were enrolled into this study. Forty age- and sex-matched volunteers (20 males and 20 females) from physical examination centers were enrolled as the control group. The inclusion criteria were as follows: (1) Patients aged 18-75 years; (2) The diagnosis of T2DM and DKD complied with the criteria reported previously (17, 18). (3) No additional intake of BCAA supplements. The exclusion criteria were as follows: liver disease, other renal diseases, heart disease, rheumatism, cancer, infectious disease, or other endocrine diseases (except diabetes mellitus). In addition, patients using drugs that affect glucose metabolism (except anti-diabetic drugs), such as glucocorticoids, or drugs that affect urine albumin creatine ratio (UACR), such as ACE inhibitors or angiotensin receptor blockers, were excluded. The clinical characteristics of all subjects are shown in **Table 1**.

**Table 1:** Clinical characteristics of patients with T2DM and the controls.

	Control group (n=40)	DM group (n=43)	DKD group-1 (n=47)	DKD Group-2 (n=30)	p-value
Gender (female/male)	20/20	21/22	21/26	13/17	0.267
Age (year)	59.63 ± 6.21	60.23 ± 9.83	57.43 ± 12.11	61.5 ± 9.45	0.292
Duration (year)	–	9.95 ± 7.72	12.38 ± 7.60	17.57 ± 7.82**^#&^**	**<0.001**
BMI (kg/m^2^)	25.62 ± 2.58	25.75 ± 3.89	27.57 ± 3.82*^#^	27.68 ± 4.56*^#^	**0.017**
WHR	0.90 ± 0.05	0.91 ± 0.07	0.94 ± 0.07*^#^	0.94 ± 0.07*	**0.008**
FPG (mmol/L)	4.93 ± 0.69	8.32 ± 2.79*	9.61 ± 3.54*	7.60 ± 2.79*	**<0.001**
Hb (g/L)	148.12 ± 17.04	144.65 ± 17.46	142.87 ± 19.95	127.27 ± 20.14*^#&^	**<0.001**
Alb (g/L)	45.16 ± 4.17	43.29 ± 3.74*	44.31 ± 4.15	38.107 ± 4.79*^#&^	**<0.001**
Urea (mmol/L)	4.99 ± 1.07	5.56 ± 1.37	6.12 ± 2.00*	8.99 ± 4.35*#&	**<0.001**
Cr (μmol/L)	81.32 ± 13.15	64.02 ± 16.14*	74.38 ± 24.98	123.46 ± 79.93*#&	**<0.001**
eGFR	80.74 ± 14.33	95.54 ± 15.18*	95.38 ± 32.17*	65.31 ± 33.95**^#&^**	**<0.001**
UA (μmol/L)	331.73 ± 61.59	344.53 ± 102.71	360.38 ± 81.55	371.01 ± 65.87	0.176
TG (mmol/L)	1.57 ± 0.69	2.01 ± 1.05	2.32 ± 1.61*	2.37 ± 2.06*	0.057
TC (mmol/L)	5.25 ± 0.91	4.98 ± 1.30	4.92 ± 1.26	5.28 ± 1.66	0.452
HDL-C (mmol/L)	1.37 ± 0.30	1.17 ± 0.28*	1.06 ± 0.19*	1.20 ± 0.29	**<0.001**
LDL-C (mmol/L)	3.30 ± 0.74	3.38 ± 0.99	3.41 ± 1.07	3.53 ± 1.13	0.822
UACR (mg/g)	6.46 (4.89,10.48)	7.78 (5.60,11.90)	67.91*^#^ (43.25,116.76)	963.56*^#&^ (611.51,6375.06)	**<0.001**
24-h UMA (mg)	9.35 (8.08,12.66)	13.60 (10.58,19.24)	103.50*^#^ (55.30,148.78)	1160.14*^#&^ (690.24, 1887.34)	**<0.001**
24-h TP (g)	0.04 (0.03,0.06)	0.08* (0.05,0.10)	0.14*^#^ (0.22,0.3)	2.26*^#&^ (1.35,3.74)	**<0.001**

BMI, body mass index; WHR, waist to hip ratio; HbA1c, glycated hemoglobin; FPG, fasting plasma glucose; FINS, fasting insulin; Hb, hemoglobin; Alb, albumin; Cr, Creatinine; eGFR, estimated glomerular filtration rate; UA, uric acid; TG, triglyceride; TC, total cholesterol; HDL-C, high-density lipoprotein cholesterol; LDL-C, low density lipoprotein cholesterol; NAG, N-acetyl-beta-amino glucosidase; UACR, urinary albumin to creatinine ratio; 24-h UMA, 24h urine microalbumin; 24-h TP, 24h urinary total protein.

*means compared with the control group, p < 0.05;

# means compared with the normal proteinuria group, p<0.05;

^&^means compared with microalbuminuria group, p < 0.05.

The bold values denote statistical significance at P < 0.05 level.

Patients were divided into three groups according to UACR: DM group (normal proteinuria, UACR < 30 mg/g, n = 43), DKD group-1 (microalbuminuria, 30 ≤ UACR ≤ 300 mg/g, n = 47), and DKD group-2 (macroalbuminuria groups, UACR > 300 mg/g, n = 30). This study was approved by the Ethics Review Committee of Chu Hsien-I Memorial Hospital of Tianjin Medical University and in accordance with the Helsinki Declaration.

### 2.2 Laboratory examination

All participants fasted at 10:00 pm; venous blood and morning urine were collected at 8:00 am the next day. Blood samples were allowed to stand at room temperature for 30 minutes and then centrifuged at 3500 g/min for 15 minutes. The top layer was taken after centrifugation and serum was used to detect several factors, including blood glucose metabolism indicators: fasting plasma glucose (FPG), fasting insulin, fasting C-peptide, and glycohemoglobin; blood routine: hemoglobin (Hb); blood lipid metabolism indicators: high-density lipoprotein cholesterol(HDL-C), low-density lipoprotein cholesterol(LDL-C), triglyceride (TG), total cholesterol (TC); liver function: alanine aminotransferase (ALT), aspartate aminotransferase (AST), and albumin(Alb); renal function: blood urea nitrogen (BUN), creatinine (Cr), estimated glomerular filtration pass rate (eGFR), uric acid (UA); urine biochemistry: UACR, urine microalbumin (UMA), 24-hour total urine protein(24-h TP). The remaining serum samples were stored at -80°C until the final analysis.

BCAA and BCKA levels in patient serum were determined using liquid chromatography-mass spectrometry (LC-MS). The steps are as follows: (1) Remove the previously collected serum sample stored in a -80°C freezer, thaw at 4°C, transfer 10 μL to a 1.5 mL enzyme-free EP tube and place the sample on ice. (2) Add 5 μL of the internal standard mixture and 100 μL of a 1:1 mixture of ethacrynic acid and methanol pre-cooled at -20°C. (3) Pre-cool the shaker to 4°C and vortex the EP tubes as described above for 5 min. (4) Centrifuge the EP tubes at 14,000g for 15 min in a centrifuge at 4°C. (5) Transfer the supernatant from the EP tube to a new 1.5 mL enzyme-free EP tube and blow dry with nitrogen (if not measured immediately after blowing dry with nitrogen, store temporarily in a -80°C freezer). (6) Add 100 μL of 20% methanol to the EP tube to redissolve the sample and vortex it for 2 min. (7) Place the EP tube in a 4°C centrifuge at 14,000g for 5 min. (8) Transfer the supernatant to a supersampling vial, taking care that no air bubbles appear during the process, and inject the sample *via* LC-MS for detection.

Insulin resistance index (19) HOMA-IR=FBG*FINS/22.5

### 2.3 Statistical analysis

All experimental data were sorted and analyzed using IBM SPSS version 26 (V26.0, IBM Corp, Chicago, USA). Before statistical analysis, the data were tested for normal distribution and homogeneity of variance The mean ± standard deviation was used for data with a normal distribution. In contrast, differences are expressed as medians and interquartile ranges for non-normally distributed data. One-way analysis of variance (ANOVA) was used to compare the means of multiple groups with normal distribution, whereas the rank sum test was used to compare the means of multiple groups with non-normal distribution. Data were log10 transformed where necessary. Spearman’s rank correlation was used to examine the relationship between serum BCAA levels and clinical indicators in patients with T2DM. All tests were two-tailed, and statistical significance was set at *p* < 0.05.

## 3 Results

### 3.1 Clinical characteristics of patients with T2DM and healthy controls

The clinical characteristics of T2DM patients and the control group are comprehensively detailed in **Table 1**. Gender, age, uric acid, TC, TG, and LDL-C exhibited no statistically significant differences among the four distinct groups (*p* > 0.05). Compared to the control group, DKD group-1 showed higher BMI, WHR, FPG, Urea, eGFR, TC, and HDL-C levels. Moreover, UACR, 24-h UMA, and 24-h TP levels were significantly elevated in DKD group-1 compared to both the control and DM groups (*p* < 0.001). DKD group-2 had a longer disease duration, higher BMI, WHR, Urea, Cr, UACR, 24-hour UMA, and 24-hour TP levels compared to the other groups. However, FPG, Hb, Alb, and eGFR levels were lower in DKD group-2 relative to the other groups.

These comprehensive clinical characterizations underscore the distinctions in various parameters across different groups, emphasizing the dynamic nature of clinical characteristics in T2DM and DKD progression.

### 3.2 Concentrations of BCAA and BCKA in the serum of patients with T2DM and healthy controls

**Figure 1:**
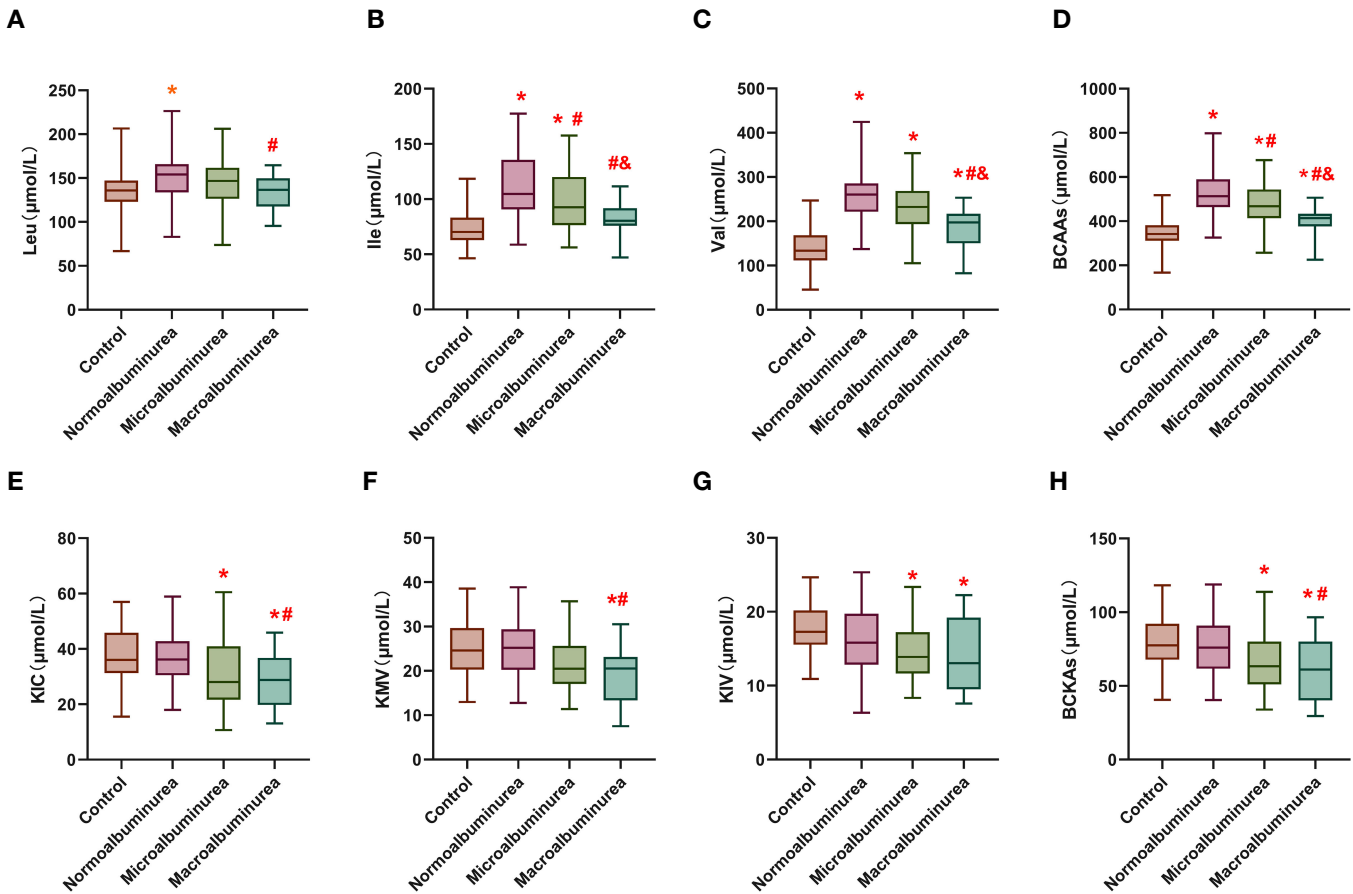
Serum levels of BCAA and BCKA in different groups. **(A–C)** are leucine, isoleucine and valine concentrations in the serum of the different groups. **(D)** is the total branched-chain amino acids concentration in the serum of the different groups. **(E–G)** are α-Cketoisohexanoic aci, α-vketoisovaleric acid and α-Keto-β-methylpentanoic acid concentrations in the serum of the different groups. **(H)** is the total branched-chain keto acids concentration in the serum of the different groups. Leu: Leucine; Ile: Isoleucine; Val, Valine; KIC, α-Cketoisohexanoic acid; KIV, α-vketoisovaleric acid; KMV, α-Keto-β-methylpentanoic acid. BCAA, Total branched-chain amino acids; BCKA, Total branched-chain keto acids. *means compared with the control group, *p* < 0.05; ^#^means compared with the normal proteinuria group, *p*<0.05; ^&^means compared with microalbuminuria group, *p* < 0.05.

We employed LC-MS for the quantification of serum levels of BCAA comprising leucine, isoleucine, and valine, as well as BCKA encompassing KIC, KIV and KMV. The serum levels of BCAA and BCKA across the four distinct groups are graphically depicted in **Figure 1**, with detailed data presented in **Table S1**. Notably, the serum BCAA levels exhibited a significant elevation in the DM group (527.75 ± 120.18 μmol/L) in comparison to the control group (345.98 ± 67.35 μmol/L). Moreover, a noteworthy observation emerged: BCAA levels already exhibited a reduction at the stage of microproteinuria (DKD group-1) in contrast to the DM group, with a progressive decline in BCAA levels noted with the advancement of DKD.

In contrast, serum BCKA levels did not display a significant disparity between the DM group (76.92 ± 19.06 μmol/L) and the healthy control group (79.85 ± 17.80 μmol/L). However, there was a discernible decrease in BCKA levels observed in DKD group-1 (69.78 ± 24.29 μmol/L), with a more pronounced decline evident in DKD group-2 (62.53 ± 23.53 μmol/L).

These findings shed light on the dynamic alterations in serum BCAA and BCKA levels across different stages of DKD and highlight the potential significance of these metabolites as biomarkers in the context of diabetes-related renal dysfunction.

### 3.3 Relationship between serum levels of BCAA and BCKA and clinical indicators in patients with T2DM

**Figure 2:**
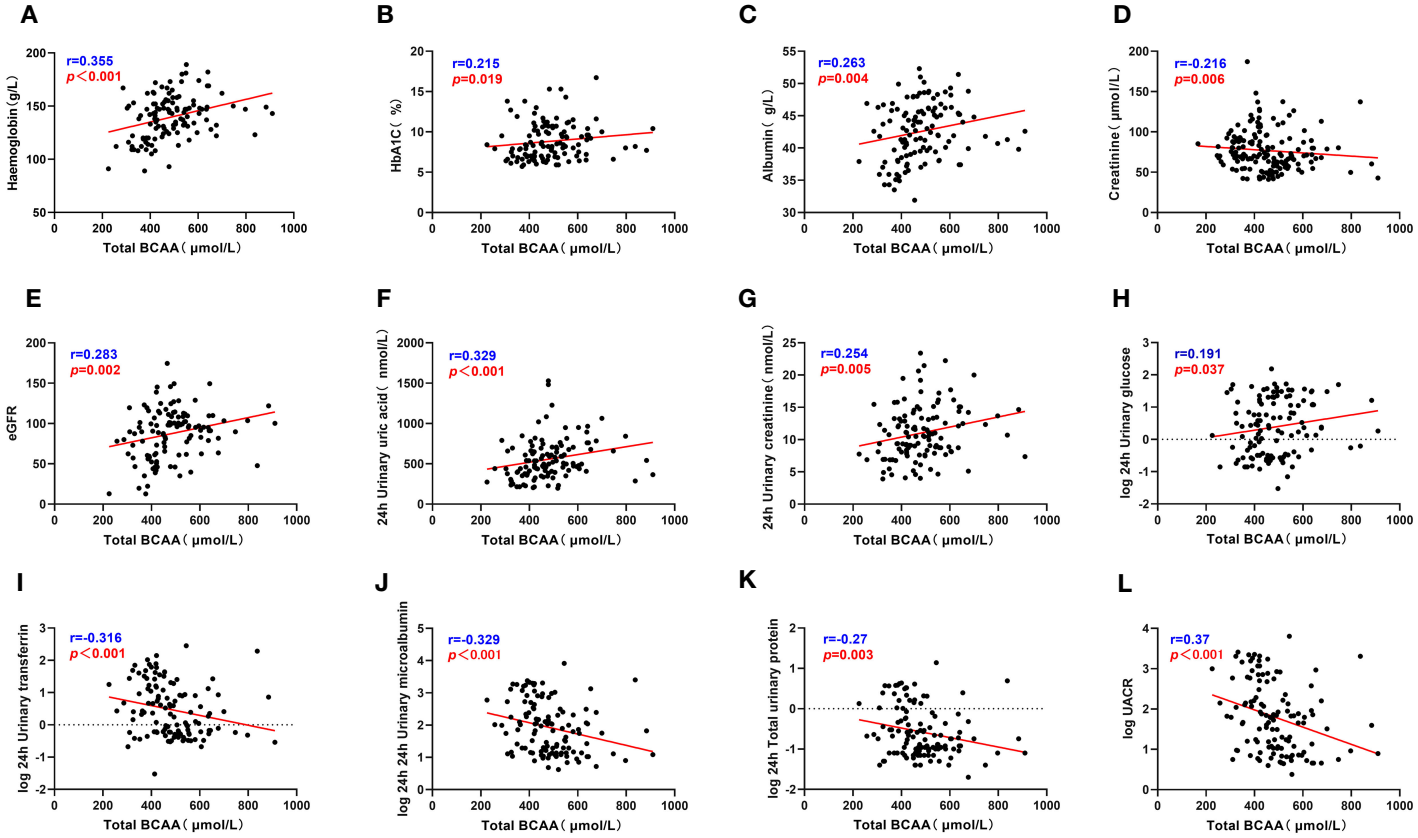
Total BCAA correlate with clinical indicators. **(A–L)** describes the correlation between total BCAA and hemoglobin, glycated hemoglobin, serum albumin, serum creatinine, eGFR, 24h uric acid, 24h urine creatinine, 24h urine glucose, 24h urine transferrin, 24h urine microalbumin, 24h total urine protein and UACR respectively. *“r”* represents the correlation coefficient, *p*<0.05 is considered statistically different.

Correlation analysis was used to examine the relationship between serum BCAA and BCKA levels and various clinical parameters among patients with T2DM (**Table S2**). Serum BCAA levels exhibited positive correlations with parameters such as HbA1c, Hb, Alb, ESR, eGFR, 24-hour UA, 24-hour urine glucose, and 24-hour urine creatinine levels, indicating that higher BCAA levels were associated with higher values of these clinical parameters. Conversely, negative correlations were identified between serum BCAA levels and UACR, Cr, TRF, IgG, 24-hour UMA, and 24-hour TP levels, implying that elevated BCAA levels were linked to lower values of these clinical markers. These findings are visually represented in **Figure 2** and detailed in **Table S2**.

**Figure 3:**
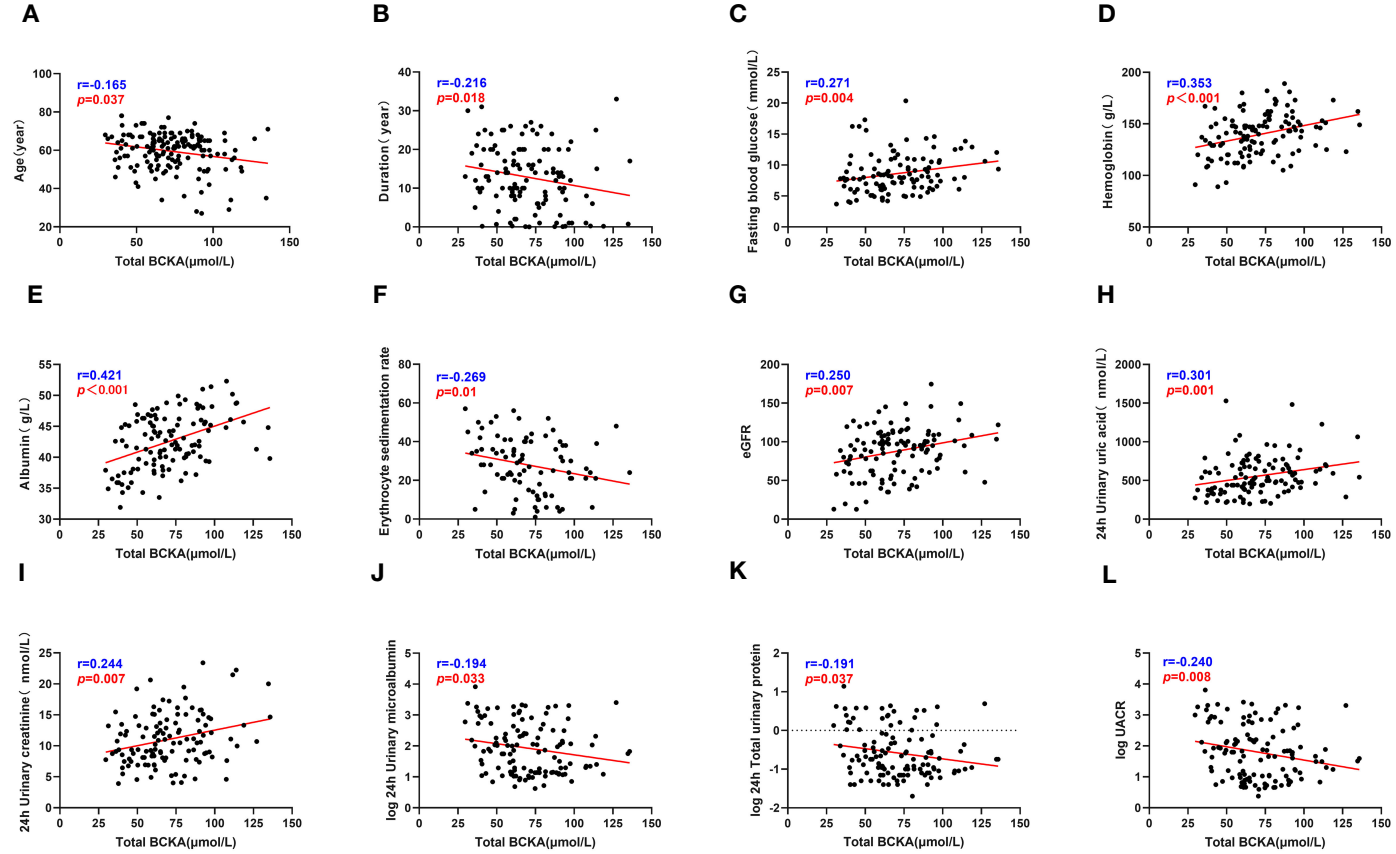
Total BCKA correlate with clinical indicators. **(A–L)** describes the correlation between total BCKA and age, duration, fasting blood glucose, hemoglobin, serum albumin, ESR, eGFR, 24h uric acid, 24h urine creatinine, 24h urine microalbumin, 24h total urine protein and UACR respectively; *“r”* represents the correlation coefficient, *p*<0.05 is considered statistically different.

The correlation analysis revealed significant associations between serum BCKA levels and several clinical parameters among patients with T2DM. Specifically, BCKA demonstrated positive correlations with FPG, Hb, Alb, ESR, eGFR, 24-hour uric acid, and 24-hour urine creatinine levels, indicating that higher BCKA levels were associated with elevated values of these clinical markers. Conversely, negative correlations were observed between BCKA levels and age, diabetes duration, IgG, 24-hour UMA, and 24-hour TP levels, suggesting that increased BCKA levels were linked to lower values of these parameters. These findings are visually represented in **Figure 3** and detailed in **Table S2**.

Collectively, the data within this group emphasize the intricate relationship between glycemic control, nutritional status, and renal function in individuals with T2DM and their influence on serum BCAA and BCKA levels.

### 3.4 Multiple linear regression analysis of serum BCAA and BCKA

In our analysis, we utilized BCAA as the dependent variable and incorporated various patient-specific factors, including age, diabetes duration, Hb, Alb, HbA1c, eGFR, TRF, UACR, UMA, and 24-hour urinary creatinine, to establish a comprehensive multivariate regression equation (as presented in **Table 2**).Consequently, our analysis unveiled statistically significant associations between BCAA and different levels of Alb (β = 4.088, t = 2.003, *p* = 0.048), TRF (β = -1.350, t = -3.449, *p* = 0.001), 24-h UMA (β = -73.861, t = -4.011, *p* < 0.001), and 24-hour urinary creatinine (β = 7.954, t = 3.262, *p* = 0.016). These results underscored the independent influence of Alb, TRF, UMA, and urinary creatinine levels on BCAA concentrations. These findings are visually represented in **Figure 3** and detailed in **Table S2**.

**Table 2:** Multiple regression analysis of influencing factors of BCKA.

Variable	β	*t*	*p-*value
Age	0.103	0.398	0.692
Gender	-0.212	-0.044	0.965
Duration	-0.106	-0.382	0.704
HbA1C	1.068	1.148	0.253
Alb	1.088	2.928	**0.004**
eGFR	0.054	0.660	0.511
TRF	-0.180	-2.461	**0.015**
24-h UMA	-7.296	-2.139	**0.035**
24-h UCr	1.681	2.426	**0.017**

β represents the regression coefficient; t represents the results of a t-test on the regression coefficients; p<0.05 is considered statistically different.

The bold values denote statistical significance at P < 0.05 level.

Similarly, we employed BCKA as the dependent variable to construct multivariate regression equations (outlined in **Table 3**). Our analysis indicated statistically significant relationships between BCKA levels and varying Alb levels (β = 1.088, t = 2.928, *p* = 0.004), TRF levels (β = -0.18, t = -2.461, *p* = 0.015), UMA levels (β = -7.296, t = -2.139, *p* = 0.035), and urinary creatinine levels (β = 1.681, t = 2.426, *p* = 0.017). These findings demonstrate that Alb, TRF, UMA and urinary creatinine levels all serve as independent influencing factors for BCKA concentrations.

**Table 3:** Multiple regression analysis of influencing factors of BCAA.

Variable	β	*t*	*p-*value
Age	1.014	0.743	0.459
Duration	2.594	1.779	0.078
HbA1C	6.930	1.433	0.155
Hb	0.384	0.574	0.567
Alb	4.088	2.003	**0.048**
eGFR	0.399	0.881	0.380
TRF	-1.350	-3.449	**0.001**
24-h UMA	-73.861	-4.011	**<0.001**
24-h UCr	7.954	2.438	**0.016**

β represents the regression coefficient; t represents the results of a t-test on the regression coefficients; p<0.05 is considered statistically different.

The bold values denote statistical significance at P < 0.05 level.

Our comprehensive multivariate regression analysis reveals a significant association between these clinical parameters of T2DM patients and the levels of BCAA/BCKA, particularly the renal function indicators.

### 3.5 Correlation of BCAA and BCKA with clinical indicators in patients of different genders with T2DM

In the subsequent analysis, we proceeded to stratify all patients diagnosed with T2DM based on gender, followed by an examination of the relationship between serum BCAA and BCKA levels and various clinical indicators, as presented in **Table S3**.

Among the male cohort, a noteworthy positive correlation was observed between BCAA levels and FPG (r = 0.411, *p* = 0.001) as well as HOMA-IR (r = 0.292, *p* = 0.02). Intriguingly, no such correlation was identified among their female counterparts. Furthermore, within the female subgroup, BCAA and BCKA exhibited a more pronounced positive correlation with Hb levels in comparison to their male counterparts. Conversely, in males, BCAA and BCKA displayed a stronger positive correlation with Alb levels compared to females. Shifting our focus to liver function indicators, ALT and AST displayed no significant association with BCAA levels in males; however, they exhibited a robust correlation among female patients. These findings align closely with those reported in a prior study examining alterations in serum BCAA levels among individuals afflicted with Non-Alcoholic Fatty Liver Disease (NAFLD) (20).Additionally, we explored urinary renal function-related markers, namely RBP, TRF, IgG, UACR, and UMA. Strikingly, these indicators exhibited significant associations with BCAA levels in both male and female patients. Notably, the correlation coefficients were notably higher among the female cohort.

In summary, our gender-stratified analysis revealed distinct patterns of correlation between serum BCAA and BCKA levels and various clinical indicators in male and female T2DM patients. These findings underscore the importance of considering gender-specific variations in the metabolic profile of T2DM patients, providing valuable insights into potential avenues for personalized therapeutic interventions.

## 4 Discussion

In this study, we investigated the changes in serum BCAA and BCKA levels in patients with T2DM; our results showed that serum BCAA levels were significantly higher in the diabetic group than in the healthy controls, consistent with previously reported studies (21). In addition, a longitudinal study of adult health in Brazil (14) showed that higher levels of BCAA were independently predictive of diabetes. A study of serum amino acid measurements in patients with T2DM also found that ketogenic amino acid levels were higher than normal in patients with diabetes (22). This shows that there is a close relationship between BCAA and T2DM, with abnormalities in the catabolism of BCAA in patients with T2DM causing an increase in serum BCAA concentrations.

Patients with T2DM are usually associated with insulin resistance. Studies have shown (23, 24) that insulin resistance can reduce the activity of the branched-chain α-keto acid dehydrogenase complex in the liver and adipose tissue, inhibiting the catabolism of BCAA. Furthermore, the chronic elevation of BCAA can contribute to the development of insulin resistance in humans (25). In addition, reduced uptake of BCAA by skeletal muscle due to reduced insulin action can also increase blood levels of BCAA (25). A recent study also showed that genes involved in BCAA catabolism are suppressed in the skeletal muscles of insulin-resistant individuals (26). Elevated levels of BCAA in insulin-resistant populations has been suggested to be associated with a gut microbiome rich in BCAA biosynthesis (27). Therefore, the elevated BCAA levels found in the sera of individuals with T2DM may result from a combination of these factors.

Taken together, the elevated levels of BCAA can promote insulin resistance and diabetes progression, and previous studies have suggested that BCAA can be used as a biomarker for a variety of diseases including diabetes mellitus (14), heart failure (16), and cancer (15). Therefore, we initially speculated that elevated BCAA might act as a biomarker for the progression of DKD, but our study found that even in microproteinuria, BCAA was decreased compared to the normo-proteinuria group, which was contrary to our expectations. As protein intake did not change significantly in patients at this stage of the disease, we hypothesized that there were abnormalities in the excretion and reabsorption of BCAA in the kidneys of patients at the microproteinuria stage, leading to a decrease in BCAA levels despite no change in intake, further suggesting that BCAA may be an indicator of kidney damage. However, due to technical limitations, we lack supporting evidence for BCAA levels in the above patients, which will need to be refined in subsequent studies.

Our results showed that serum BCAA concentrations gradually decreased as the degree of proteinuria progressed in patients with DKD, which has been verified in previous studies (28). We consider that this may be due to the accumulation of uremic toxins in the patient’s body, decreased appetite, and inadequate nutritional intake of the patient. On the other hand, patients with DKD develop acid-base imbalance and metabolic acidosis is sufficient to promote the oxidative breakdown of renal BCAA by affecting the activity of the key enzyme of BCAA catabolism-BCKDH complex (29). In addition, it has been suggested that inflammation in patients with chronic kidney disease can lead to decreased plasma amino acid concentrations (30).

Similarly, we observed that the serum BCAA levels of the patients started to decrease in the microproteinuria stage compared to those in the T2DM group. The serum BCAA levels decreased further when progressing to the massive proteinuria stage. Our results showed that serum BCAA levels were negatively correlated with urinary microalbumin levels and positively correlated with Alb levels. This shows that BCAAs are closely associated with the nutritional status of patients with T2DM. The blood Alb level is an important indicator of the body’s nutritional status. Studies have reported that leucine, a BCAA, can promote Alb synthesis in rat primary hepatocytes by activating the mTOR signal transduction pathway (31–33). In patients with DKD, as the degree of proteinuria increases, the blood Alb level decreases and the BCAA is more of a compensatory synthetic protein, allowing the serum BCAA level to decrease.

Urinary TRF is a negatively-charged, medium-molecule protein that is synthesized primarily by hepatocytes in the human body for iron transport (34, 35). TRF does not normally pass freely across the glomerular filtration barrier and is present in very small amounts in the urine. If the glomerular filtration barrier is damaged, urinary TRF can pass through, entering the urine and causing proteinuria. This property makes urinary TRF an indicator of early kidney damage (36). In a study of five early biomarkers for predicting DKD, urinary TRF was significantly associated with the development of DKD and could be used to assess early glomerular damage in patients with diabetes and effectively predict the development of early DKD (37). Our study found that serum BCAA and TRF levels were negatively correlated in patients with T2DM with different stages of proteinuria. Multifactorial linear regression analysis showed that serum TRF was an independent influencing factor for BCAA and BCKA. In contrast, UMA was independently associated with serum BCAA levels in patients with T2DM. In summary, serum BCAA levels may help predict early DKD.

Our study has some limitations. First, the study was a single-center, cross-sectional study, and the role of changes in serum BCAA levels in the progression of DKD requires further prospective cohort studies with larger samples to confirm our findings. Second, due to technical limitations that we are working to resolve, our study has not yet been refined enough to determine urinary BCAA concentrations in the patients in our study.

## 5 Conclusion

Our study revealed that serum BCAA levels were notably elevated in individuals diagnosed with T2DM compared to the levels observed in the general population. Serum BCAA levels decreased and correlated with indicators of renal function with the progression of DKD. These findings suggest that the concentration of serum BCAA in patients with T2DM may serve as a significant predictive marker for the development of DKD.

## Data availability statement

The raw data supporting the conclusions of this article will be made available by the authors, without undue reservation.

## Ethics statement

The studies involving humans were approved by Tianjin Medical University Chu Hsien-l Memorial Hospital. The studies were conducted in accordance with the local legislation and institutional requirements. The participants provided their written informed consent to participate in this study.

## Author contributions

ML: Writing – original draft. YY: Writing – original draft. YL: Data curation, Methodology, Investigation, Writing – original draft. XP: Formal Analysis, Investigation, Writing – original draft. YH: Data curation, Writing – original draft. XZ: Formal Analysis, Methodology, Writing – original draft. HS: Writing – review & editing. CS: Writing – review & editing.
