# Supplementary material for: Serum branched chain amino acids: an effective indicator of diabetic kidney disease
Source: Front Endocrinol (Lausanne). 2023 Nov 27;14:1269633. doi: 10.3389/fendo.2023.1269633 (PMC10711269; doi:10.3389/fendo.2023.1269633)
Supplement: Supplementary file 1 [file Table_1.docx]

**Table S1. Serum levels of BCAA and BCKA in patients with T2DM and the controls**

|  | Control group  (n=40） | DM group  （n=43） | DKD group-1（n=47） | DKD group-2  （n=30） | ANOVA  *p-*value |
| --- | --- | --- | --- | --- | --- |
| Leu  (μmol/L） | 134.27±25.44 | 155.02±31.93**^*^** | 149.62±37.50 | 138.42±27.55^#^ | **0.012** |
| Ile  (μmol/L） | 72.83±15.83 | 113.31±32.90**^*^** | 99.34±26.70**^*#^** | 87.22±21.74**^#$^** | **＜0.001** |
| Val  (μmol/L） | 138.88±42.78 | 259.42±70.85**^*^** | 232.76±61.10**^*^** | 203.66±70.30**^*#$^** | **＜0.001** |
| KIC  (μmol/L） | 37.74±9.63 | 36.59±9.93 | 32.76±13.83^*^ | 28.99±11.66**^*#^** | **0.007** |
| KIV  (μmol/L） | 17.69±6.29 | 15.47±5.04 | 14.50±4.92**^*^** | 14.35±5.67**^*#^** | **0.001** |
| KMV  (μmol/L） | 24.42±6.19 | 24.86±6.55 | 22.51±7.41^*^ | 19.18±6.75**^*^** | **0.003** |
| BCAA  (μmol/L） | 345.98±67.35 | 527.75±120.18**^*^** | 481.72±117.72**^*#^** | 429.30±108.84**^*#$^** | **＜0.001** |
| BCKA  (μmol/L） | 79.85±17.80 | 76.92±19.06 | 69.78±24.29^*^ | 62.53±23.53**^*#^** | **0.004** |

Note: Leu: Leucine; Ile: Isoleucine; Val: Valine; KIC: α-Cketoisohexanoic acid; KIV: α-vketoisovaleric acid; KMV: α-Keto-β-methylpentanoic acid.

BCAA: Total branched-chain amino acids; BCKA: Total branched-chain keto acids.

* means compared with the control group, *p* < 0.05;

# means compared with the DM group, *p*＜0.05;

& means compared with DKD group-1, *p* < 0.05.

**Table S2． Spearman rank correlation analysis between serum total BCAA and BCKA levels and clinical parameters in patients with T2DM**

|  | Total BCAA | | Total BCKA | |
| --- | --- | --- | --- | --- |
|  | *r* | *p-*value | *r* | *p-*value |
| Age | -0.134 | 0.091 | -0.165 | **0.037** |
| Duration | -0.129 | 0.159 | -0.216 | **0.018** |
| BMI | -0.009 | 0.921 | -0.058 | 0.535 |
| HbA1C | 0.215 | **0.019** | 0.147 | 0.112 |
| FPG | 0.144 | 0.133 | 0.271 | **0.004** |
| Hb | 0.355 | **＜0.001** | 0.353 | **＜0.001** |
| Alb | 0.263 | **0.004** | 0.394 | **＜0.001** |
| ESR | -0.320 | **0.002** | -0.269 | **0.010** |
| Cr | -0.216 | **0.006** | -0.047 | 0.558 |
| eGFR | 0.283 | **0.002** | 0.250 | 0.007 |
| 24-h UA | 0.329 | **＜0.001** | 0.301 | ＜0.001 |
| 24-h UGlu | 0.191 | **0.037** | 0.161 | 0.079 |
| 24-h UCr | 0.254 | **0.005** | 0.244 | **0.007** |
| log TRF | -0.316 | **＜0.001** | -0.174 | 0.058 |
| log IgG | -0.342 | **＜0.001** | -0.204 | **0.026** |
| log24-h UMA | -0.334 | **＜0.001** | -0.194 | **0.033** |
| log 24-h UTP | -0.270 | **0.003** | -0.191 | **0.037** |
| log UACR | -0.37 | **＜0.001** | -0.240 | **0.008** |

Note：“*r*”represents the correlation coefficient; *p*<0.05 is considered statistically different.

**Table S3. Correlation of BCAA and BCKA with clinical indicators in DM patients of different genders**

|  | Male  （n=65） | | | | Female  （n=55） | | | |
| --- | --- | --- | --- | --- | --- | --- | --- | --- |
|  | BCAA | | BCKA | | BCAA | | BCKA | |
|  | *r* | *p*-value | *r* | *p*-value | *r* | *p*-value | *r* | *p*-value |
| **FPG** | 0.179 | 0.156 | 0.411 | **0.001** | 0.061 | 0.683s | 0.101 | 0.501 |
| **HOMA-IR** | -0.037 | 0.775 | 0.292 | **0.020** | 0.069 | 0.653 | 0.029 | 0.848 |
| **Hb** | 0.291 | **0.019** | 0.280 | **0.024** | 0.343 | **0.011** | 0.405 | **0.002** |
| **CRP** | -0.020 | 0.883 | 0.312 | **0.016** | -0.022 | 0.878 | -0.253 | 0.079 |
| **Alb** | 0.251 | **0.044** | 0.438 | **＜0.001** | 0.230 | 0.092 | 0.371 | **0.005** |
| **ALT** | 0.004 | 0.977 | 0.251 | **0.043** | 0.205 | 0.134 | 0.326 | **0.015** |
| **AST** | 0.035 | 0.781 | 0.184 | 0.142 | 0.176 | 0.204 | 0.332 | **0.014** |
| **Urea** | -0.252 | **0.043** | -0.026 | 0.838 | -0.098 | 0.481 | -0.025 | 0.856 |
| **eGFR** | 0.257 | **0.038** | 0.204 | 0.103 | 0.267 | 0.056 | 0.273 | 0.050 |
| **NAG** | -0.026 | 0.838 | -0.083 | 0.510 | -0.303 | **0.025** | -0.020 | 0.887 |
| **RBP** | -0.289 | **0.020** | -0.310 | **0.012** | -0.415 | **0.002** | -0.087 | 0.527 |
| **log TRF** | -0.250 | **0.044** | -0.228 | 0.068 | -0.408 | **0.002** | -0.083 | 0.547 |
| **log IgG** | -0.245 | **0.049** | -0.203 | 0.105 | -0.446 | **0.001** | -0.171 | 0.213 |
| **log UACR** | -0.295 | **0.017** | -0.290 | **0.019** | -0.465 | **＜0.001** | -0.177 | 0.197 |
| **log 24-h UMA** | -0.271 | **0.029** | -0.274 | **0.027** | -0.424 | **0.001** | -0.108 | 0.433 |
| **log 24-h UTP** | -0.215 | 0.086 | -0.266 | **0.032** | -0.330 | **0.014** | -0.095 | 0.491 |

Note：“*r*” represents the correlation coefficient; p<0.05 is considered statistically different.
